# Associations between Fat Mass and Fat Free Mass with Physical Fitness in Adolescent Girls: A 3-Year Longitudinal Study

**DOI:** 10.3390/biology11050783

**Published:** 2022-05-21

**Authors:** Mario Kasović, Ana Oreški, Tomáš Vespalec, Marta Gimunová, Lovro Štefan

**Affiliations:** 1 Department of General and Applied Kinesiology, Faculty of Kinesiology, University of Zagreb, 10 000 Zagreb, Croatia; mario.kasovic@kif.hr; 2 Department of Sport Motorics and Methodology in Kinanthropology, Faculty of Sports Studies, Masaryk University, 62 500 Brno, Czech Republic; vespalec@fsps.muni.cz (T.V.); gimunova@fsps.muni.cz (M.G.); 3 Secondary School ‘Gospodarska Škola Varaždin’, 42 000 Varazdin, Croatia; ana.oreski2@gmail.com; 4 Department of Research and Examination (RECETOX), Faculty of Science, Masaryk University, 611 37 Brno, Czech Republic

**Keywords:** body composition, performance, youth, follow-up, linear mixed models

## Abstract

**Simple Summary:**

During the adolescent period, the associations between body composition and health-related physical fitness have been less studied. By examining such associations, health-related and physical education professionals will be able to monitor and track both components and their inter-correlation characteristics. Therefore, the main purpose of this study was to examine the longitudinal associations between the two components of body composition (namely fat mass and fat free mass) and physical fitness performance tests. In 240 adolescent girls, the findings showed that fat mass was inversely associated with standing broad jump, sit-ups in 60 s, and squats in 60 s, while positive associations with the 800 m and the 400 m run were observed. Fat free mass was positively associated with standing broad jump, sit-ups in 60 s, and squats in 60 s, while negative associations with the 800 m and the 400 m run were found. Fat mass and fat free mass seem to be similarly, but oppositely associated with physical fitness tests in adolescent girls. Thus, interventions that target lower fat mass and higher fat free mass values in order to improve physical performance should be advocated.

**Abstract:**

The main purpose of the study was to examine the longitudinal associations between fat mass and fat free mass with health-related physical fitness. Two-hundred and forty 15-year old adolescent girls were measured at the baseline and after a period of 3 years (17 years). Health-related physical fitness included the following tests: (1) explosive power of the lower extremities (standing broad jump); (2) muscle endurance of the trunk (sit-ups in 60 s); (3) flexibility (sit-and-reach test); (4) muscle endurance of the lower extremities (squats in 60 s); (5) aerobic endurance (the 800 m run test); and (6) speed endurance (the 400 m running test). Fat mass and fat free mass were assessed using the bioelectrical impedance method. Longitudinal associations were analyzed with linear mixed model estimates. After adjusting for body mass index, fat mass was negatively associated with standing broad jump (*β =* −1.13, *p* < 0.001), sit-ups in 60 s (*β =* −0.27, *p* < 0.001), and squats in 60 s (*β =* −0.27, *p* < 0001), while positive associations with the 800 m running test (*β =* 0.02, *p* < 0.001) and the 400 m running test (*β =* 0.02, *p* < 0.001) were observed. On the other hand, fat free mass was positively associated with standing broad jump (*β =* 1.14, *p* < 0.001), sit-ups in 60 s (*β =* 0.28, *p* < 0.001), and squats in 60 s (*β =* 0.28, *p* < 0001), while the 800 m running test (*β =* −0.02, *p* < 0.001) and the 400 m running test (*β =* −0.02, *p* < 0.001) exhibited negative associations. This study shows that fat mass and fat free mass components are longitudinally, but oppositely associated with health-related physical fitness in adolescent girls.

## 1. Introduction

Excessive fat mass in youths has become a serious global public health concern in the past two decades [1]. Estimates suggest that between 20% and 45% of children are overweight and obese [2], and rising trends have been observed in both developed and less developed countries [3]. According to the World Health Organization [4], the highest prevalence of overweight and obesity has been observed in southern European and Mediterranean countries. In Croatia, recent findings have shown that the prevalence of overweight and obesity in childhood and adolescence is between 30% and 45%, which is higher compared to regional estimates [5]. Being overweight and obese from an early age may lead to negative health-related outcomes in later life including a higher incidence of cardiovascular and metabolic diseases [6] and premature mortality rates [7].

Higher levels of body fatness may impact the physical fitness in children and adolescents [8,9,10,11,12,13,14,15,16]. Specifically, previous evidence has shown that excessive fat mass or body mass index values have been associated with poor cardiorespiratory fitness [13,14] and worse performance in the coordination and agility tests (the 4 × 10 m shuttle run test) [13,14], jumping tests (standing broad jump) [12,13,14], and motor fitness (the 40 m test) [12]. On the other hand, a higher fat free mass has been positively associated with handgrip strength [12,13,15]. Previous research has shown that bioelectrical impedance analysis is one of the most common instrumentations to assess the body composition in children and adolescents [16,17,18]. For example, a systematic review by Talma et al. [16] showed good intraclass reliability correlations of ≥0.82, while the test–retest mean differences ranged between 7.5% and 13.4%, indicating a considerable measurement error. Another systematic review showed that the best method for bedside assessment was bioelectrical impedance, and although it has limitations, further development of body composition methods may provide promising results in obtaining the body composition in youths [17,18]. Although an effort to examine the associations between body composition with other health-related physical fitness components has been made, most of the previous research in older children has been cross-sectional [12,13,14,15,19], with no obvious direct associations. It has been argued that physical fitness may be seen as a marker of adiposity, considering the body mass index as an independent variable [20]. Furthermore, very little is known as to whether such associations are present during adolescence. In terms of body composition and physical fitness, a most recent study conducted among Croatian youth showed that the body mass index increased, while the level of coordination/agility, flexibility, and cardiorespiratory performance decreased in the past two decades, which was especially visible in adolescent girls [21]. Since physical fitness is a powerful marker of health [22] and tracks well from childhood to adulthood [23,24,25], it is necessary to establish whether body composition (fat mass and fat free mass) is significant and longitudinal predictors of other health-related physical fitness components.

Therefore, the main purpose of the study was to examine the longitudinal associations between fat mass and fat free mass with health-related physical fitness in adolescent girls. We hypothesized that the fat mass and fat free mass would exhibit similar, yet opposite associations with physical fitness over a 3-year follow-up period.

## 2. Materials and Methods

### 2.1. Study Participants

For the purpose of this 3-year longitudinal study, we recruited adolescent girls measured at the ages of 15 years and 17 years. Of the ten high schools in the city of ‘Varaždin’, three were randomly selected by the randomization process and removing school codes from a box. In that way, all schools had the same probability to be selected. In each school, a total of four classes were selected by using a random sampling approach, which gave 286 adolescent girls in total. Forty-six of them were excluded from further analyses (i.e., 19 girls were absent when the study was conducted and 37 were measured at only 1 time-point). Thus, 240 adolescent girls were enrolled in further statistical analyses. By using a two-tailed linear multiple regression test for longitudinal data, the effect size of 0.05, a statistical significance of *p* < 0.05, a power of 0.95, and the number of predictors being set at 1, the appropriate sample size was estimated as N = 262. Before the study, all parents and participants had provided their written informed consent to enter the study. All of the analyses and procedures during the study were anonymous, following the principles of the Declaration of Helsinki [26]. The Ethical Committee of the Faculty of Kinesiology, University of Zagreb (Croatia) approved of the study.

### 2.2. Body Composition

To measure the body composition, we used bioelectrical impedance analysis (Omron BF500 Body Composition Monitor, Omron Medizintechnik, Vernon Hills, IL, USA). The methodology of using the device has been described previously [27]. In brief, the participant stands barefoot on a metal footpad while holding a pair of electrodes attached to a handle with arms extended in front of the chest. ***Fat mass*** and ***fat free mass*** were predicted with the manufacturer’s pre-programmed equations including sex, age, stature, and body weight. The participants performed the test three times to assess the level of the internal consistency. The reliability coefficient for the three measurements was beyond 0.90. Before the testing procedure in the morning hours between 7:00 and 10:00 h, each participant was instructed not to consume food or water. Standing height and weight were measured using Seca portable 202 scales (Seca, Hamburg, Germany) and a digital scale (Seca, model 769).

### 2.3. Health-Related Physical Fitness

Health-related physical fitness included motor and functional abilities measured by the following performance tests: (1) explosive power of the lower extremities (standing broad jump); (2) muscle endurance of the trunk (sit-ups in 60 s); (3) flexibility (sit-and-reach test); (4) muscle endurance of the lower extremities (squats in 60 s); (5) Aerobic endurance (the 800 m running test); and (6) speed endurance (the 400 m running test). The testing methodology for each performance test has previously been described in detail [28,29,30,31].

### 2.4. Data Analysis

The data normality was calculated using the Kolmogorov–Smirnov test. Since all variables were normally distributed, we applied the arithmetic mean and standard deviation (SD) to present the results. The differences between the baseline and follow-up values were calculated by the Student *t*-test for dependent samples. In order to examine the magnitude of change measured at two time-points, the Cohen *d* effect size (ES) was calculated with the following classification: (i) <0.2 (trivial); (ii) 0.2–0.5 (moderate); (iii) 0.5–0.8 (large); and (iv) >0.8 (very large) [32]. Linear mixed models were used to analyze the longitudinal associations between the fat mass and fat free mass with the physical fitness performance tests. The results were expressed by *β* coefficients with 95% confidence intervals (95% CI) using 500 cluster bootstrap samples to account for the dependence between the repeated measures. To account for repeated measures, a first-order autoregressive correlation structure was used. The first model was unadjusted and the second one was adjusted for the body mass index. Two-sided *p*-values were used, and significance was set at α < 0.05. All of the analyses were calculated in the Statistical Packages for Social Sciences v.23 (SPSS, Chicago, IL, USA).

## 3. Results

The raw data and the basic descriptive statistics of this study, as presented in Table 1, were used in a previously published paper on the same topic [33]. Mean and SD are shown in Table 1. The magnitude of the effect sizes ranged from small to large (i.e., height and weight exhibited the smallest effect sizes, while the largest effect sizes for aerobic capacity, speed endurance and flexibility were observed). Gradually, smaller effect sizes could be observed for the muscle endurance of the lower extremities, muscle endurance of the trunk, body composition, and the explosive power of the lower extremities.

Longitudinal associations between the fat mass and health-related physical fitness components are presented in Table 2. In the unadjusted models, the strongest associations were observed between the fat mass and standing broad jump, followed by sit-ups in 60 s, squats in 60 s, the sit-and-reach test, the 800 m running test, and the 400 m running test. When each model was adjusted for the body mass index, similar associations and the same order between the fat mass with other health-related physical fitness components were obtained.

Table 3 shows the longitudinal associations between the fat free mass and health-related physical fitness components. Higher levels of fat free mass in a mixed model design was associated with the standing broad jump, followed by sit-ups in 60 s, squats in 60 s, the sit-and-reach test, the 800 m running test, and the 400 m running test. When the models were adjusted for the body mass index, a similar association remained.

Of note, the Pearson coefficient of correlation showed that the fat mass was inversely associated with the standing broad jump (*r* = −0.65, *p* < 0.001), sit-ups in 60 s (*r* = −0.31, *p* < 0.001), and the number of squats in 60 s (*r* = −0.35, *p* < 0.001), while there were positive associations with the sit-and-reach test (*r* = 0.12, *p* = 0.008), the 800 m running (*r* = 0.43, *p* < 0.001), and the 400 m running (*r* = 0.51, *p* < 0.001) tests. The same associations between the fat free mass with other health-related physical fitness components were observed, only in a different direction.

## 4. Discussion

The main purpose of the study was to examine the longitudinal associations between the fat mass and fat free mass with other health-related physical fitness components in adolescent girls. The main findings were: (1) During the follow-up period, the largest declines in aerobic and speed endurances were observed, followed by flexibility, muscle endurance of the lower extremities and muscle endurance of the trunk, and (2) the fat mass and fat free mass appeared to have opposite longitudinal and significant associations with physical fitness, even after adjusting for body mass index.

Our findings extend and agree with the previous observations conducted among adolescents [12,13,14,15]. Specifically, a longitudinal study by Zaqout et al. [12] showed that the child’s body mass index was a significant determinant of physical fitness, where the strongest and negative body mass index effects were seen in the cardiorespiratory fitness and the strength of the lower extremities, but an advantageous effect on handgrip strength was observed. When the fat mass and fat free mass were derived from skinfold thicknesses, underweight girls achieved better results in the bend arm hang test, but both underweight boys and girls performed lower in handgrip strength compared with normal weight children [13]. In the same study, overweight and obese adolescents presented lower performance in cardiorespiratory fitness, muscle endurance, explosive power of the lower extremities, and coordination/agility, but a higher performance in handgrip strength [13]. Health-related physical fitness has also been associated with the total body fat obtained from dual energy X-ray absorptiometry and Bod Pod air displacement plethysmography and central fat calculated from the waist circumference [14]. All components of physical fitness including muscular strength, cardiorespiratory fitness, and speed/agility have shown negative associations with all markers of total and central body fat in adolescent boys and girls, even after adjusting for age, pubertal status, and objectively measured physical activity [14]. Finally, increasing the body weight status had a negative association with measures of strength that involve lifting the body, but is associated with improved performances on tests using flexion (handgrip strength) and extension (knee) as primary movements [15]. Similar associations have been confirmed in the previous literature on younger children [19,20,34,35]. In studies conducted among preschoolers, a higher fat mass was associated with lower cardiorespiratory fitness, agility, and the explosive power of the lower extremities [16,17].

In order to improve the physical fitness, an overweight and obese lifestyle at young ages should be targeted [12]. Overweight and obese children and adolescents have been recommended to start participating in regular physical activity by doing static strength exercises [36]. Fitter individuals are more prone to possess higher levels of fat free mass compared to their unfit counterparts [14]. The mechanism underlying the association between the body composition and physical fitness is related to muscle metabolism, where the energy expenditure can adequately enhance large muscle groups. Thus, individuals who are fitter may have less fat mass. It should be highlighted that physical fitness can be an important marker of health at a given age and later in the future [22], irrespective of physical activity. However, both the physical activity and body composition have been proposed as considerations when designing special interventions and policies to enhance physical fitness [12].

## 5. Limitations

This study had several limitations. First, the follow-up period seemed to be somewhat shorter. Second, by examining the associations between the body composition and physical fitness performance only in both boys and girls, we might have obtained different results comparable between the sexes. Third, no information regarding the frequency, intensity, nor duration of physical activity and maturational level were collected, which could have influenced the associations. Fourth, the criterion and convergent validity properties and measurement errors for the bioelectrical impedance were considered in the previous research when compared with dual X-ray absorptiometry [16]. Thus, it is possible that the fat mass and fat free mass results obtained in our study might be different from the ‘true’ values. Finally, we were unable to analyze the raw data for the resistance, reactance, and phase angle using a bioelectrical impedance spectroscopy analyzer (model 4200, Xitron Technologies, San Diego, CA, USA) [37].

## 6. Conclusions

This study shows that the fat mass and fat free mass may serve as significant longitudinal predictors for health-related physical fitness. Higher levels of fat mass are associated with lower cardiorespiratory, muscular, speed, and motor performance, even after adjusting for body mass index. On the other hand, adolescent girls with higher fat free mass levels exhibited better physical fitness scores in each component.

## Figures and Tables

**Table 1:** Descriptive statistics of the study participants at the baseline and follow-up (N = 240).

Study Variables	Baseline Mean (SD)	Follow-Up Mean (SD)	∆ (%)	ES	*p*-Value
**Stature (cm)**	165.8 (7.3)	167.6 (7.5)	1.1	0.24	<0.001
**Weight (kg)**	60.2 (14.0)	62.3 (14.8)	3.5	0.15	<0.001
**Fat mass (%)**	27.0 (10.5)	29.8 (10.1)	10.4	0.27	<0.001
**Fat free mass (%)**	73.0 (10.5)	70.2 (10.1)	−3.8	0.27	<0.001
**Standing broad jump (cm)**	178.5 (28.3)	170.9 (28.1)	−4.3	−0.27	<0.001
**Sit-ups (reps in 60 s)**	55.4 (11.0)	51.2 (10.1)	−7.6	−0.40	<0.001
**Sit-and-reach test (cm)**	69.4 (8.9)	64.5 (9.6)	−7.1	−0.53	<0.001
**Squats (reps in 60 s)**	48.7 (8.0)	44.9 (7.9)	−7.8	−0.48	<0.001
**The 800 m running test (min)**	4.49 (0.78)	4.99 (0.78)	11.1	0.64	<0.001
**The 400 m running test (min)**	1.47 (0.31)	1.64 (0.38)	11.6	0.49	<0.001

*p* < 0.05.

**Table 2:** The mixed model estimates of the association between fat mass with other physical fitness components (N = 240).

Study Variables	Fat Mass (%)					
Physical Fitness Components	β	95% CI	Std. Error	df	*t*-Value	*p*-Value
**Standing broad jump (cm)**						
*Unadjusted*	−1.09	−1.26–−0.91	0.09	448.92	−12.24	<0.001
*Adjusted*	−1.13	−1.33–−0.92	0.10	379.08	−10.91	<0.001
**Sit-ups (reps in 60 s)**						
*Unadjusted*	−0.27	−0.38–−0.17	0.05	263.83	−5.01	<0.001
*Adjusted*	−0.27	−0.44–−0.10	0.09	330.85	−3.14	0.002
**Sit-and-reach test (cm)**						
*Unadjusted*	0.10	0.01–0.20	0.05	368.53	2.10	0.037
*Adjusted*	0.12	−0.02–−0.25	0.07	471.45	1.73	0.084
**Squats (reps in 60 s)**						
*Unadjusted*	−0.23	−0.31–−0.15	0.04	273.43	−5.60	<0.001
*Adjusted*	−0.27	−0.40–−0.15	0.06	357.90	−4.29	<0.001
**The 800 m running test (min)**						
*Unadjusted*	0.03	0.02–0.04	0.00	282.09	6.93	<0.001
*Adjusted*	0.02	0.01–0.03	0.01	375.50	3.25	<0.001
**The 400 m running test (min)**						
*Unadjusted*	0.02	0.01–0.03	0.00	307.46	8.83	<0.001
*Adjusted*	0.02	0.01–0.02	0.00	425.81	6.33	<0.001

Abbreviations: CI—Confidence interval, Std. Error—Standard error, df—degrees of freedom. Data were analyzed with the use of a separate physical fitness component being associated with fat mass. Unadjusted model: Each physical fitness component was entered separately into the model. Adjusted model: Each physical fitness component was entered separately into the model and adjusted for the body mass index. *p* < 0.05.

**Table 3:** The mixed model estimates of the association between the fat free mass with other physical fitness components (N = 240).

Study Variables	Fat Free Mass (%)					
Physical Fitness Components	β	95% CI	Std. Error	df	*t*-Value	*p*-Value
**Standing broad jump (cm)**						
*Unadjusted*	1.10	0.92–1.27	0.09	452.35	12.39	<0.001
*Adjusted*	1.14	0.94–1.34	0.10	383.17	11.07	<0.001
**Sit-ups (reps in 60 s)**						
*Unadjusted*	0.28	0.17–0.38	0.05	266.44	5.12	<0.001
*Adjusted*	0.28	0.11–0.44	0.08	334.31	3.28	<0.001
**Sit-and-reach test (cm)**						
*Unadjusted*	−0.10	−0.20–−0.01	0.05	370.25	−2.05	0.041
*Adjusted*	−0.11	−0.24–0.02	0.07	474.98	−1.67	0.095
**Squats (reps in 60 s)**						
*Unadjusted*	0.23	0.15–0.31	0.04	275.66	5.70	<0.001
*Adjusted*	0.28	0.15–0.40	0.06	361.52	4.42	<0.001
**The 800 m running test (min)**						
*Unadjusted*	−0.03	−0.04–−0.02	0.00	283.84	−7.01	<0.001
*Adjusted*	−0.02	−0.03–−0.01	0.01	378.63	−3.33	<0.001
**The 400 m running test (min)**						
*Unadjusted*	−0.02	−0.02–−0.01	0.00	309.72	−8.93	<0.001
*Adjusted*	−0.02	−0.02–−0.01	0.00	429.53	−6.46	<0.001

Abbreviations: CI—Confidence interval, Std. Error—Standard error, df—degrees of freedom. Data were analyzed with the use of a separate physical fitness component being associated with fat free mass. Unadjusted model: Each physical fitness component was entered separately into the model. Adjusted model: Each physical fitness component was entered separately into the model and adjusted for the body mass index. *p* < 0.05.

## Data Availability

The data that support the findings of this study are available upon reasonable request from the corresponding author. The data are not publicly available due to privacy or ethical restrictions.
